# High disease activity persists in adults with childhood-onset lupus

**DOI:** 10.1136/lupus-2026-002138

**Published:** 2026-08-05

**Authors:** Rachel Koelmeyer, Kate Gregory, Rangi Kandane-Rathnayake, Fiona Goldblatt, Sean O’Neill, Maureen Rischmueller, Mandana Nikpour, Geraldine Hassett, Pravin Hissaria, Dwarakanathan Ranganathan, Claire Barrett, Ashleigh Hennessey, Ted Tsai, Peter Gowdie, Eric F Morand, Alberta Hoi

**Affiliations:** 1 Centre for Inflammatory Diseases, Monash University, Clayton, Victoria, Australia; 2 The University of Auckland, Auckland, Auckland, New Zealand; 3 Department of Medicine, Monash University, Clayton, Victoria, Australia; 4 Flinders Medical Centre, Bedford Park, South Australia, Australia; 5 Northern Clinical School, The University of Sydney Faculty of Medicine and Health, Sydney, New South Wales, Australia; 6 The Queen Elizabeth Hospital, Woodville South, South Australia, Australia; 7 University of Sydney, Sydney, New South Wales, Australia; 8 Royal Prince Alfred Hospital, Camperdown, New South Wales, Australia; 9 Liverpool Hospital, Liverpool, New South Wales, Australia; 10 Royal Adelaide Hospital, Adelaide, South Australia, Australia; 11 The University of Adelaide, Adelaide, South Australia, Australia; 12 Griffith University, Brisbane, Queensland, Australia; 13 Redcliffe Hospital, Redcliffe, Queensland, Australia; 14 Royal Brisbane and Women’s Hospital, Herston, Queensland, Australia; 15 Canberra Hospital, Garran, Australian Capital Territory, Australia; 16 Monash Children’s Hospital, Clayton, Victoria, Australia; 17 Sub-Faculty of Clinical and Molecular Medicine, Monash University, Clayton, Victoria, Australia; 18 Rheumatology, Monash Health, Clayton, Victoria, Australia; 19 Medicine, Monash University Faculty of Medicine Nursing and Health Sciences, Clayton, Victoria, Australia; 20 Monash Health, Clayton, Victoria, Australia

**Keywords:** Lupus Erythematosus, Systemic, Epidemiology, Lupus Nephritis

## Abstract

**Objective:**

Childhood-onset SLE (cSLE) is often described as more severe than adult-onset disease (aSLE) based largely on cross-sectional studies. We examined differences in disease characteristics, medication use and long-term outcomes between cSLE and patients with aSLE in adulthood using longitudinal data from a national cohort to address this knowledge gap.

**Methods:**

A retrospective cohort study was conducted using the Australian Lupus Registry and Biobank. Data included demographics, disease duration, autoantibody profile, classification criteria, time-adjusted mean SLE Disease Activity Index 2000 (SLEDAI-2K AMS), SLE Damage Index (SDI) and SF36 health-related quality of life data. Key outcomes were AMS and time in the Lupus Low Disease Activity State (LLDAS) and damage accrual. Bivariate tests were used for group comparisons.

**Results:**

Of 519 patients with SLE enrolled between 2011 and 2022 with ≥12 months of data available, 68 (13%) had cSLE. Median (IQR) age at enrolment was 39 (30–51) years; 88.1% female; majority were of white or Asian ethnicity. At baseline, more patients with cSLE had damage (SDI≥1) (53% vs 40%, p=0·05) and renal involvement (62% vs 37%, p<0·001). Over median (IQR) follow-up of 5 (2·6–9·3) years, patients with cSLE had higher disease activity (median (IQR) AMS 5·0 (3·0–6·4) cSLE vs 3·6 (1·9–5·1) aSLE, p<0.001), were more likely to be in High Disease Activity Status (SLEDAI-2K ≥10 ever) and were less likely to achieve LLDAS-50 (36% vs 51%, p=0·036). Flare rates, damage accrual and quality of life during follow-up were similar between groups.

**Conclusions:**

Adults with cSLE entered adult follow-up with higher baseline damage and continued to experience a higher longitudinal disease activity burden than patients with aSLE. These findings highlight the importance of early recognition, consistent longitudinal monitoring and timely escalation of therapy during earlier years of disease to reduce long-term disease burden.

**WHAT IS ALREADY KNOWN ON THIS TOPIC**

- Previous studies comparing childhood-onset SLE (cSLE) to adult-onset SLE (aSLE) have indicated potential differences in organ involvement, such as more frequent renal or haematological disease in cSLE.
- However, little is known about the long-term disease course and other longitudinal disease outcomes in these patients.

**WHAT THIS STUDY ADDS**

- This multicentre study is the first to show that time-adjusted disease activity in patients with cSLE remains high well into adulthood.
- At enrolment, a greater proportion of patients with cSLE had renal involvement compared with those with aSLE. Over time, however the overall pattern of organ involvement converged with that observed in aSLE.
- Despite this, the median time adjusted mean SLE Disease Activity Index-2000 remained significantly higher in the cSLE group, and these patients were markedly less likely to sustain Lupus Low Disease Activity State during their follow-up.

**HOW THIS STUDY MIGHT AFFECT RESEARCH, PRACTICE OR POLICY**

- Persistently high disease activity in patients with cSLE extending into adulthood highlights the need for closer disease monitoring and early sustained control.
- Although the rate of damage accrual appears similar between cSLE and aSLE, patients with cSLE begin adulthood with a higher baseline burden of irreversible damage.
- This suggests that early, proactive, treat-to-target management is essential to prevent further accumulation of damage. Strengthening transitional care and addressing unmet therapeutic needs should also be prioritised to improve long-term outcomes for this population.

## Introduction

 SLE is a multisystem autoimmune disease, with 10%–20% of cases diagnosed during childhood (<18 years).^1 2^ Childhood-onset SLE (cSLE) is associated with a more severe disease phenotype than adult-onset SLE (aSLE), characterised by a higher frequency of renal, neurological and haematological involvement,^3–10^ as well as higher levels and greater seropositivity for autoantibodies, such as anti-dsDNA,^6 8 9 11 12^ anti-Sm and anticardiolipin antibodies.^13^ While numerous studies have demonstrated that cSLE is characterised by greater disease activity compared with aSLE, few have examined how disease activity evolves as patients with cSLE transition into adulthood.^8^ Existing research on cSLE in adulthood is largely cross-sectional, with limited longitudinal analyses, leaving it unclear whether the aggressive disease activity observed in childhood persists over time.^4 5 8^ This study aims to determine whether patients with cSLE continue to exhibit higher disease activity than those with aSLE, and whether differences in organ manifestations remain between the two groups in adulthood.

## Methods

### Study design

Retrospective cohort study.

### Patients and setting

Patients with cSLE (diagnosis prior to age 18 years) or aSLE (diagnosis at or after age 18 years) were identified from the Australian Lupus Registry and Biobank (ALRB).^14^ To be enrolled in the ALRB, patients must meet either the 1997 American College of Rheumatology (ACR)^15^ or the 2012 Systemic Lupus International Collaborating Clinics (SLICC)^16^ classification criteria. The ALRB is a multicentre longitudinal registry enrolling patients with SLE from public tertiary care sites across Australia, with a standardised prospective collection of demographic, clinical, treatment, disease activity, organ damage and patient-reported outcome data. For this study, data were included from nine active Australian sites, and we included all patients enrolled between 2007 and 2022 with at least 12 months of ALRB data and a minimum of two visits. All participants provided written informed consent for collection and use of their data for this research.

### Data collection

Baseline data collected included gender, self-reported race/ethnicity, age at diagnosis and enrolment, autoantibodies, comorbidities and social history including employment and educational status. Significant health events, hospitalisations, medication use and disease activity measures including SLE Disease Activity Index 2000 (SLEDAI-2K), physician global assessment and Safety of Estrogens in Lupus Erythematosus, National Assessment (SELENA/SLEDAI) Flare Index were recorded during routine clinic reviews. Disease activity over time was calculated using the adjusted mean SLEDAI (AMS) score, defined as the area under the curve of SLEDAI-2K scored over time.^17^ This was determined by adding the area of each visit interval block and dividing by the total length of time for the entire observational period. High Disease Activity Status (HDAS) was identified if the patient had an ever attainment of SLEDAI-2K ≥10.^18 19^ Low disease activity was determined using the Lupus Low Disease Activity State (LLDAS) definition.^20^ Flares were recorded using the SELENA/SLEDAI Flare Index during routine clinic reviews and categorised as mild/moderate or severe.^21^ Annualised flare rates were calculated as the number of recorded flares during the observation period divided by each patient’s duration of follow-up. Lupus-related damage was assessed at baseline and annually using SLICC/ACR Damage index (SDI).^22^ Additionally, health-related quality of life was assessed annually using the SF-36 (36-Item Short Form Health Survey) mental component summary and physical component summary (MCS and PCS, respectively).^23^

### Statistical analysis

Patient characteristics were described at enrolment using appropriate summary statistics, that is, median (IQR) for continuous variables and frequency (%) for categorical variables. These characteristics were compared between patients with cSLE and patients with aSLE using bivariate tests. These tests included χ^2^ tests to compare proportions and Wilcoxon rank-sum tests to compare medians. Group differences were considered to be statistically significant at a value of p<0.05. All analyses were performed using Stata statistical software (V.18). Regression modelling with appropriate adjustment for confounding by time since diagnosis and age was performed to look for association with cSLE and longitudinal outcomes.

### Patient and public involvement

No patients nor the general public were involved in the design, conduct or interpretation of the findings. However, patients participating in the ALRB receive regular updates regarding research studies conducted via the ALRB through an annual newsletter.

### Role of the funding source

No funding bodies had a role in the study design; collection, analysis and interpretation of the data; drafting of the manuscript, or the decision to submit the work for publication.

## Results

A total of 519 patients from nine active sites were included in this study; 68 (13%) were patients with cSLE and 451 (87%) patients with aSLE. All patients were over 18 years old at the time of enrolment. The median follow-up duration was 4.8 years (IQR: 2.6–9.3 years), with no significant difference between the groups: patients with cSLE had a median observation time of 4.2 years (IQR: 2.1-9.9) while patients with aSLE had 4.9 years (IQR: 2.7-9.2). Patients with cSLE were adults at enrolment, with a median age of 25 years (IQR: 20–33.5), which was significantly younger than the median age of aSLE (41 years; IQR: 32–53, p<0.001).

Disease duration at enrolment was longer for patients with cSLE with a median 13.2 years (IQR: 7.3–20.6 years) compared with 4.2 years (IQR: 0.8–11.8 years) for patients with aSLE (p<0.001). Self-reported race/ethnicity differed numerically between groups, with a higher proportion of Asian patients in the cSLE group and a higher proportion of White patients in the aSLE group, although the overall difference did not reach statistical significance. Specifically, 47.1% (32/68) of patients with cSLE were Asian, compared with 35.7% (161/451) of patients with aSLE. Similarly, also 47.1% (32/68) of cSLE were White compared with 53% (239/451) of patients with aSLE, although this difference did not reach statistical significance (see table 1). The “Other” category included patients reporting ancestry not captured by the predefined registry categories; the numbers are too small for reliable subgroup analysis.

**Table 1:** Patient characteristics

Number (%) unless otherwise specified	Total study population (n=519)	Childhood-onset SLE (n=68)	Adult-onset SLE (n=451)	Evidence of a difference by age at diagnosis* (P value)
Age at diagnosis (years), median (IQR)	29 (22–42)	14 (12–15)	32 (25–44)	<0.001
Age at enrolment (years), median (IQR)	39 (30–51)	25 (20–33.5)	41 (32–53)	<0.001
Disease duration at enrolment (years), median (IQR)	5.9 (1.2–13.3)	13.15 (7.3–20.6)	4.2 (0.8–11.8)	<0.001
Female	457 (88.1%)	56 (82.4%)	401 (88.9%)	0.12
Ethnicity				
White	271 (52.2%)	32 (47.1%)	239 (53.0%)	0.17
Asian	193 (37.2%)	32 (47.1%)	161 (35.7%)	
Other ethnicity	42 (8.1%)	3 (4.4%)	39 (8.6%)	
Unknown	13 (2.5%)	1 (1.5%)	12 (2.7%)	
Family history of SLE	50 (9.6%)	11 (16.2%)	39 (8.6%)	0.084

* Based on appropriate bivariate test (χ2 test, Fisher’s exact test, t test or Wilcoxon rank-sum test).

Organ involvement at baseline based on ACR and/or SLICC criteria revealed that patients with cSLE had significantly more renal involvement than patients with aSLE, with 61.8% (42/68) of patients with cSLE having a history of renal involvement compared with 37.3% (168/451) of patients with aSLE (p<0.001). Frequencies of other manifestations, such as haematological and neuropsychiatric disease, were comparable between the groups. Baseline serology showed higher rates of positive double-stranded deoxyribonucleic acid (dsDNA) antibodies in patients with cSLE (85.3%; 58/68) compared with patients with aSLE (74.3%; 335/451), although this difference was not statistically significant (see table 2).

**Table 2:** Disease manifestations and damage present at baseline (time of enrolment into the Australian Lupus Registry and Biobank)

	Total study population (n=519)	Childhood-onset SLE (n=68)	Adult-onset SLE (n=451)	Evidence of a difference by age at diagnosis* (P value)
Disease manifestations†				
Haematological	299 (57.6%)	44 (64.7%)	255 (56.5%)	0.20
Immunological	519 (100.0%)	68 (100.0%)	451 (100.0%)	
Mucocutaneous	398 (76.7%)	58 (85.3%)	340 (75.4%)	0.072
Musculoskeletal	401 (77.3%)	52 (76.5%)	349 (77.4%)	0.87
Neuropsychiatric	70 (13.5%)	13 (19.1%)	57 (12.6%)	0.14
Renal	210 (40.5%)	42 (61.8%)	168 (37.3%)	<0.001
Serositis	156 (30.1%)	21 (30.9%)	135 (29.9%)	0.87
Serology				
ANA	495 (95.4%)	66 (97.1%)	429 (95.1%)	0.75
Anti-dsDNA	393 (75.7%)	58 (85.3%)	335 (74.3%)	0.067
Anti-Sm	78 (15.0%)	14 (20.6%)	64 (14.2%)	0.20
Antiphospholipid	216 (41.6%)	27 (39.7%)	189 (41.9%)	0.79
Damage present (SDI)‡ at baseline	218 (42.0%)	36 (52.9%)	182 (40.4%)	0.050

* Based on the appropriate bivariate test (χ2test, Fisher’s exact test, t test or Wilcoxon rank-sum test).

† ACR and/or SLICC classification criteria met at enrolment.

‡ SLE Damage index. The unadjusted OR for damage at baseline in the childhood-onset versus adult-onset subgroup was 1.7 (95% CI 95% CI 1.0 to 2.8; p=0.052), which attenuated to 1.1 (95% CI 0.6 to 1.9; p=0.76) after adjusting for baseline time since diagnosis.

ACR, American College of Rheumatology; dsDNA, double-stranded deoxyribonucleic acid; SDI, SLE Damage Index; SLICC, Systemic Lupus International Collaborating Clinic; Sm, Smith.

Disease activity adjusted for time, measured using the AMS, was significantly higher in patients with cSLE, with a median AMS of 5.0 (IQR: 3.0–6.4), compared with 3.6 (IQR: 1.9–5.1) in patients with aSLE (p<0.001; see table 3). Patients with cSLE continued to have a higher AMS than patients with aSLE when AMS calculation was stratified by follow-up year (see table 4). Patients with cSLE were more likely to have HDAS, with a SLEDAI ≥10 during the observation period (58.6%; 34/68) compared with patients with aSLE (42.0%; 171/451) (p=0.017). Conversely, patients with cSLE spent less time in LLDAS, with a median of 34.7% (IQR 8.4%–57.6%) compared with 50.2% (IQR 25.8%–74.3%) in patients with aSLE (p=0.003). Despite higher baseline renal involvement in cSLE, there were no significant differences in renal disease or other types of disease activity during the observation period (see table 3). Annualised flare rates were similar between the groups, with patients with cSLE experiencing a median flare rate of 0.4 (IQR 0–0.8) compared with 0.4 (IQR 0.1–0.8) in patients with aSLE (p=0.791).

**Table 3:** Longitudinal disease activity and damage

Longitudinal outcome	Number of participants with data available	Total study population	Childhood-onset SLE (Dx<18)	Adult-onset SLE	Association of childhood-onset SLE with outcome*
Total patient observation period, median (IQR)		4.8 (2.6–9.3)	4.15 (2.1–9.9)	4.9 (2.7–9.2)	0.41
Types of disease activity during the observation period					
Constitutional symptoms (fever)	519	26 (5%)	4 (5.9%)	22 (4.9%)	0.764
Haematological	519	160 (30.8%)	21 (30.9%)	139 (30.8%)	0.992
Immunological	519	459 (88.4%)	62 (91.2%)	397 (88.0%)	0.449
Mucocutaneous	519	339 (65.3%)	45 (66.2%)	294 (65.2%)	0.873
Musculoskeletal	519	233 (44.9%)	25 (36.8%)	208 (46.1%)	0.148
Neuropsychiatric	519	42 (8.1%)	7 (10.3%)	35 (7.8%)	0.475
Ocular	519	13 (2.5%)	0 (0%)	13 (2.9%)	0.234
Renal	519	243 (46.8%)	37 (54.4%)	206 (45.7%)	0.178
Serositis	519	79 (15.2%)	9 (13.2%)	70 (15.5%)	0.625
Vasculitis	519	36 (6.9%)	3 (4.4%)	33 (7.3%)	0.607
AMS, median (IQR~)	459	3.8 (2.0–5.3)	5 (3.0–6.4)	3.6 (1.9–5.1)	<0.001
AMS >4	459	206 (44.9%)	39 (67.2%)	167 (41.7%)	<0.001
HDAS patient†	519	205 (44.1%)	34 (58.6%)	171 (42%)	0.017
Percent time in LLDAS, median (IQR)	438	48.1 (24.1–73.1)	34.7 (8.4–57.6)	50.2 (25.8–74.3)	0.0032
Time in LLDAS ≥50%	438	210 (48.0%)	17 (32.7%)	193 (50.0%)	0.019
Annualised flare rates					
Mild/moderate, median (IQR)	519	0.4 (0–0.8)	0.4 (0–0.8)	0.4 (0.1–0.8)	0.791
Severe, median (IQR)	519	0 (0–0.2)	0 (0–0.3)	0 (0–0.2)	0.24
Time-adjusted mean prednisolone dose (mg/day)	519	2.6 (0–6)	3.3 (0.3–7)	2.4 (0–6)	0.244
Concomitant IS/HCQ/GC therapy ever‡^,§^	519	308 (59.3%)	45 (66.2%)	263 (58.3%)	0.219
Damage accrual	519	179 (34.5%)	18 (26.5%)	161 (35.7%)	0.136
Last SDI Score≥1	519	296 (57%)	43 (63.2%)	253 (56.1%)	0.268
Time-adjusted mean PCS, median (IQR)	318	46 (38.3–51.1)	46.7 (40.8–50.9)	45.5 (37.6–51.1)	0.363
Time-adjusted mean MCS, median (IQR)	318	47.9 (39.9–53.4)	50.4 (40.7–54.2)	47.2 (39.9–52.9)	0.377

* Based on appropriate bivariate test (χ2 test, Fisher’s exact test, t test or Wilcoxon rank-sum test).

† Ever had a SLEDAI-2K Score ≥10.

‡ Defined as concurrent treatment with hydroxychloroquine, prednisolone and an immunosuppressant (based on the list above).

§ Included immunosuppressants include azathioprine, ciclosporin, cyclophosphamide, leflunomide, methotrexate, mycophenolate, tacrolimus, mercaptopurine, sulfasalazine, rituximab and belimumab.

AMS, time-adjusted mean SLEDAI-2K Score; GC, glucocorticoid; HCQ, hydroxychloroquine; HDAS, High Disease Activity Status; IS, immunosuppressants; LLDAS, Lupus Low Disease Activity State; MCS, Mental Component Score; PCS, Physical Component Score; SDI, SLE Damage Index; SLEDAI-2K, SLE Disease Activity Index 2000.

**Table 4:** Descriptive statistics by year of follow-up

Clinical parameter	Year 1*			Year 2*			Year 3*		
	cSLE (n=68†)	aSLE (n=451†)	P value‡	cSLE (n=58†)	aSLE (n=401†)	P value‡	cSLE (n=53†)	aSLE (n=348†)	P value‡
Number of visits per year, median (IQR)	5 (3–6)	5 (3–7)	0.1406	3 (2–5)	4 (3–6)	0.01	4 (2–5)	4 (3–6)	0.076
Experienced HDAS	24 (41.4%)	122 (30.3%)	0.089	16 (33.3%)	74 (20.8%)	0.05	14 (34.4%)	68 (21.5%)	0.177
AMS, median (IQR)	4.8 (2.3–8.4)	3.8 (2–5.9)	0.0342	5.4 (3.8–6.4)	3.5 (2–5.3)	0.001	5.7 (4.5–7.6)	3.6 (1.5–5.4)	<0.001

* To closest visit within ±90 days of end of year of follow-up.

† Number of participants with data available on parameter less than total number of participants in some cases.

‡ Based on appropriate bivariate test (chi2, Fisher’s exact test, t test or Wilcoxon rank-sum test).

AMS, time-adjusted mean SLEDAI-2K Score; aSLE, adult-onset SLE; cSLE, childhood-onset SLE; HDAS, High Disease Activity Status.

At baseline, a higher proportion of patients with cSLE had existing damage, with 52.9% (36/68) having an SDI Score ≥1 compared with 40.4% (182/451) of patients with aSLE (p=0.05). However, over the observation period, patients with cSLE did not accrue more damage than patients with aSLE. New damage accrual (increase in SDI ≥1) was observed in 26.5% (18/68) of patients with cSLE compared with 35.7% (161/451) of patients with aSLE (p=0.136). By the end of the study, 63.2% (43/68) of patients with cSLE and 56.1% (253/451) of patients with aSLE had lupus-related damage, indicated by an SDI Score ≥1 (p=0.268). The prevalence of different types of damage accrual at baseline or during the observation period (online supplemental table 2) did not appear to differ markedly; however, statistical comparisons were not done due to lack of power to assess domain-specific damage accrual differences between patients with cSLE and patients with aSLE. The unadjusted OR for damage at baseline was 1.7 (95% CI, 95% CI 1.0 to 2.8; p=0.052), which attenuated to 1.1 (95% CI 0.6 to 1.9; p=0.76) after adjusting for time since diagnosis.

Patients with cSLE were found to be on similar or slightly higher doses of prednisolone compared with patients with aSLE (p=0.244). Rates of immunosuppressant use were comparable between the groups, with 76.5% (52/68) of patients with cSLE and 74.3% (335/451) of patients with aSLE having ever used an immunosuppressant (p=0.699). Similarly, 66.2% (45/68) of patients with cSLE and 58.3% (263/451) of patients with aSLE had been on concomitant immunosuppressant, hydroxychloroquine and glucocorticoid therapy, defined as prednisolone, hydroxychloroquine and one additional immunosuppressant (p=0.219). Examination of immunosuppressant use in the cSLE and aSLE cohorts (online supplemental table 1) demonstrated significant differences in prescribing patterns. Compared with the aSLE cohort, patients with cSLE were less likely to receive methotrexate (p=0.01) and more likely to receive mycophenolate (p=0.002). This pattern is consistent with the greater burden of renal disease observed at baseline in the cSLE cohort.

Health-related quality of life over the observation period, measured using time-adjusted mean SF-36 component scores (time-adjusted mean PCS and time-adjusted mean MCS), was similar between groups. Median time-adjusted mean PCS was 46.7 in cSLE and 45.5 in aSLE, and median time-adjusted mean MCS was 50.4 in cSLE and 47.2 in aSLE, with no statistically significant differences between the groups (table 3).

## Discussion

In this retrospective cohort study, we compared adult patients with cSLE to those with aSLE. Over a median observation period of 4.8 years, patients with cSLE demonstrated persistently higher disease activity, were more likely to experience HDAS and spent significantly less time in LLDAS. Despite higher disease activity, patients with cSLE accrued no more damage than patients with aSLE during follow-up, and the use of immunosuppressive therapies, including the concurrent use of corticosteroids, hydroxychloroquine and conventional immunosuppressants, was comparable between groups.

These findings reinforce evidence that cSLE is a more aggressive disease phenotype that remains highly active into adulthood. Previous reports have primarily relied on cross-sectional data, limiting insight into long-term disease trajectories.^13^ Our study provides longitudinal confirmation that elevated disease activity in cSLE persists well beyond the paediatric years, consistent with observations from a smaller single-centre study in Korea.^8^ The reduced cumulative time in LLDAS emphasises the difficulty of achieving sustained remission in this population and highlights the need for earlier and more targeted therapeutic intervention.

Although the proportion of patients with each manifestation ever observed during follow-up was similar between groups, these measures do not capture the persistence, recurrence, frequency or severity of activity over time. By contrast, AMS and time in LLDAS are longitudinal measures based on repeated observations. The higher AMS and lower observed time in LLDAS among patients with cSLE may therefore reflect more persistent or recurrent activity, slower resolution of active disease, or shorter periods of sustained low disease activity, rather than a substantially different spectrum of organ involvement.

Although adults with cSLE had higher disease activity across multiple longitudinal measures, this did not translate into significantly higher annualised flare rates and greater observed damage accrual during follow-up. These findings should be interpreted cautiously. High disease activity would ordinarily be expected to increase the risk of subsequent damage, and our findings do not imply that higher disease activity in cSLE is benign. Rather, much of the damage burden in adults with cSLE may have accrued before enrolment into the adult registry, consistent with their longer disease duration at baseline (tables 1 and 2). Traditional flare metrics may also be less sensitive to persistent disease activity, which in this study was captured more clearly by AMS and reduced time in LLDAS. The comparable rates of immunosuppressant use, including concomitant immunosuppressant, hydroxychloroquine and glucocorticoid therapy and the slightly higher corticosteroid exposure in patients with cSLE argue against undertreatment as an explanation for ongoing disease activity. Rather, this persistence may reflect the intrinsic immunopathology of early onset SLE, characterised by heightened immune dysregulation and longer cumulative exposure to disease processes.

We also investigated whether higher disease activity was driven by poor disease control during the transition from paediatric to adult care, a known challenge in cSLE.^24^ However, we observed persistent differences in AMS scores in patients with cSLE across the first 3 years of observation, indicating that higher disease activity is not attributable to any potential transitional care gaps.

Higher baseline damage in patients with cSLE likely reflects a combination of longer disease duration before adult registry enrolment and intrinsic differences in disease phenotype. cSLE is associated with greater genetic loading, more severe early disease, higher rates of renal and major organ involvement, and potentially greater cumulative treatment exposure, particularly glucocorticoid. These factors may contribute to damage accrual before transition to adult care. Brunner *et al* previously reported that patients with cSLE assessed during childhood had higher disease activity and accrued more damage than patients with aSLE.^4^ Similarly, a case-control study from the LUMINA cohort found higher renal damage, with a trend towards higher neuropsychiatric damage among patients with cSLE.^5^ However, findings across studies have been inconsistent, with some reports describing less damage in paediatric cohorts than adult cohorts.^7 13 25^ These differences may partly reflect variation in cohort age, disease duration and duration of follow-up, as well as recognised challenges in applying the adult version of SDI to paediatric populations.^26 27^ In our study, the median follow-up of approximately five years may not have been sufficient to capture divergence in damage scores, as the damage accrues slowly over time. Therefore the absence of greater damage accrual in the cSLE group during follow-up should be interpreted cautiously, particularly in the context of their higher damage burden at enrolment.

Notably, patients with cSLE in the ALRB cohort did not accrue damage more rapidly than those with aSLE during follow-up. With the advent of more effective treatment strategies, the capacity to limit further damage now appears comparable between patients with cSLE and patients with aSLE, offering optimism for improved long-term outcomes.

Prior studies and meta-analyses have consistently reported high renal involvement in cSLE compared with aSLE. In our cohort, renal involvement was more common at baseline among adults with cSLE, consistent with these reports.^3 4 6 8–10^ However, renal disease activity during adult follow-up did not differ significantly between groups. This distinction likely reflects differences in study design and timing. Our cohort comprised adults enrolled into an adult registry, many years after childhood diagnosis, rather than a paediatric inception cohort followed from disease onset. Renal manifestations occurring during childhood or adolescence may therefore have contributed to a higher proportion of baseline renal involvement. Unlike some published studies, we did not observe higher rates of haematological or neurological manifestations in patients with cSLE or a lower prevalence of arthritis.^5 7–10^ Instead, disease activity in patients with cSLE reflected involvement across multiple organ domains, broadly similar to patients with aSLE, suggesting a heterogenous pattern of activity rather than a single dominant manifestation.

Quality-of-life measures, including SF-36 scores, were comparable between patients with cSLE and patients with aSLE, consistent with previous studies. Although SLE is generally considered to have a substantial impact on children and adolescents, Tucker *et al* reported comparable mental and emotional functioning (MCS scores) between patients with adolescent-onset SLE and patients with aSLE, despite greater physical impacts (PCS scores) in the former group.^5^ Interestingly, patients with adolescent-onset SLE were found to have stronger social support and similar coping abilities compared with patients with aSLE. The discordance between higher disease activity and maintained quality of life scores may reflect the adaptability of patients with cSLE or limitations in capturing the full spectrum of patient-reported outcomes.

Despite being a multicentre registry, the rarity of cSLE limits the statistical power of this study, particularly for analysing less common disease manifestations. This constraint may reduce the precision of subgroup analyses and the generalisability of some findings. Race/ethnicity was captured using broad registry categories, and more granular ethnicity data, including specific Aboriginal and Torres Strait Islander, Maori/Pacific peoples and other ancestry groups, were not available for analysis. Furthermore, as most participants in the ALRB were recruited through tertiary public hospitals, the cohort may under-represent patients with milder disease or greater financial resources who receive care in the private sector. This potential selection bias should be considered when interpreting the results and their applicability to the broader SLE population.

In summary, this multicentre cohort study demonstrates that patients with cSLE continue to experience persistently higher disease activity into adulthood, yet accrue damage at a rate comparable to those with adult-onset disease. These findings suggest earlier recognition, consistent monitoring and timely escalation of therapy during the earlier years of disease can meaningfully alter long-term outcomes. Sustained implementation of treat-to-target strategies and optimised transitional care may further reduce the burden of cumulative damage in this high-risk population. Continued research into the biological drivers of persistent inflammation in cSLE is warranted to guide precision approaches that improve both disease control and quality of life across the lifespan.

## Supplementary material

10.1136/lupus-2026-002138 online supplemental file 1

## Data Availability

Data are available upon reasonable request.
