# Supplementary material for: High disease activity persists in adults with childhood-onset lupus
Source: Lupus Sci Med. 2026 Aug 5;13(2):e002138. doi: 10.1136/lupus-2026-002138 (PMC13448687; doi:10.1136/lupus-2026-002138)
Supplement: Supplementary file 1 [file lupus-13-2-s001.docx]

**Supplementary Material: High disease activity persists in adults with childhood onset lupus**

| **Supplementary Table 1: Use of immunomodulatory medicines during the observation period** | | | | |
| --- | --- | --- | --- | --- |
|  |  |  |  |  |
| **Medication use parameter** | **Total study population  (n = 519)** | **Childhood onset SLE (n = 68)** | **Adult onset SLE (n = 451)** | **Evidence of a difference by age at onset  (p-value)** |
| Ever used hydroxychloroquine | 479 (92.3%) | 66 (97.1%) | 413 (91.6%) | 0.11 |
| Ever used prednisolone | 382 (73.6%) | 54 (79.4%) | 328 (72.7%) | 0.24 |
| Ever taken an immunosuppressant other than hydroxychloroquine | 387 (74.6%) | 52 (76.5%) | 335 (74.3%) | 0.70 |
| Ever taken azathioprine | 177 (34.1%) | 23 (33.8%) | 154 (34.1%) | 0.96 |
| Ever used belimumab | 19 (3.7%) | 1 (1.5%) | 18 (4.0%) | 0.30 |
| Ever taken cyclophosphamide | 33 (6.4%) | 6 (8.8%) | 27 (6.0%) | 0.37 |
| Ever taken cyclosporin | 5 (1.0%) | 1 (1.5%) | 4 (0.9%) | 0.51 |
| Ever taken leflunomide | 23 (4.4%) | 2 (2.9%) | 21 (4.7%) | 0.52 |
| Ever taken methotrexate | 116 (22.4%) | 7 (10.3%) | 109 (24.2%) | 0.010 |
| Ever used mycophenolate | 215 (41.4%) | 40 (58.8%) | 175 (38.8%) | 0.002 |
| Ever used rituximab | 49 (9.4%) | 10 (14.7%) | 39 (8.6%) | 0.11 |
| Ever used sulfasalazine | 2 (0.4%) | 0 (0.0%) | 2 (0.4%) | 1.00 |
| Ever used tacrolimus | 19 (3.7%) | 2 (2.9%) | 17 (3.8%) | 0.73 |
| Maximum Treatment Intensity Score^#^, median (IQR) | 3 (2, 4) | 4 (2, 4) | 3 (2, 4) | 0.19 |
| Time-adjusted mean Treatment Intensity Score^#^, median (IQR) | 2.08 (1.19, 3) | 2.31 (1.43, 3) | 2.06 (1.16, 3) | 0.30 |
| Abbreviations: IQR, inter-quartile range. |  |  |  |  |
| ^#^The Treatment Intensity Score attempts to quantify the intensity of treatment used to control disease activity. Each SLE-directed immunomodulatory agent contributes 1 point to the treatment intensity score, including hydroxychloroquine, conventional immunosuppressants and biologic or advanced therapies. Glucocorticoid exposure is weighted according to daily prednisolone-equivalent dose, with 1 point assigned for doses >0 to ≤7.5mg/day, and 2 points assigned for >7.5mg/day. | | | | |

| **Supplementary Table 2: Domain-specific SLE Damage Index information** | | | |
| --- | --- | --- | --- |
|  |  |  |  |
| **Damage parameter** | **Total study population  (n = 519)** | **Childhood onset SLE* (n = 68)** | **Adult onset SLE* (n = 451)** |
| ***Baseline damage:*** |  |  |  |
| Cardiovascular | 29 (5.8%) | 2 (3.1%) | 27 (6.2%) |
| Gastrointestinal | 4 (0.8%) | 1 (1.5%) | 3 (0.7%) |
| Neuropsychiatric | 66 (13.1%) | 11 (16.9%) | 55 (12.6%) |
| Ocular | 24 (4.8%) | 3 (4.6%) | 21 (4.8%) |
| Renal | 35 (7.0%) | 8 (12.3%) | 27 (6.2%) |
| Peripheral vasculature | 25 (5.0%) | 7 (10.8%) | 18 (4.1%) |
| Pulmonary | 20 (4.0%) | 4 (6.2%) | 16 (3.7%) |
| Musculoskeletal | 60 (11.9%) | 9 (13.8%) | 51 (11.6%) |
| Skin-related | 32 (6.4%) | 6 (9.2%) | 26 (5.9%) |
| Other damage accrual | 31 (6.2%) | 4 (6.2%) | 27 (6.2%) |
| ***Damage accrual over observation period:*** | |  |  |
| Cardiovascular | 35 (6.7%) | 3 (4.4%) | 32 (7.1%) |
| Gastrointestinal | 9 (1.7%) | 0 (0.0%) | 9 (2.0%) |
| Neuropsychiatric | 25 (4.8%) | 4 (5.9%) | 21 (4.7%) |
| Ocular | 33 (6.4%) | 3 (4.4%) | 30 (6.7%) |
| Renal | 34 (6.6%) | 5 (7.4%) | 29 (6.4%) |
| Peripheral vasculature | 23 (4.4%) | 3 (4.4%) | 20 (4.4%) |
| Pulmonary | 17 (%) | 1 (%) | 16 (%) |
| Musculoskeletal | 62 (11.9%) | 7 (10.3%) | 55 (12.2%) |
| Skin-related | 32 (6.2%) | 3 (4.4%) | 29 (6.4%) |
| Other damage accrual | 30 (5.8%) | 2 (2.9%) | 28 (6.2%) |
| *Evidence of a difference by age of SLE onset not assessed due to power issues for most domains. | | | |
